# Undecidability and opacity of metacognition in animals and humans

**DOI:** 10.3389/fpsyg.2013.00171

**Published:** 2013-04-09

**Authors:** Kevin B. Clark, Derrick L. Hassert

**Affiliations:** ^1^Research and Development Service, Veterans Affairs Greater Los Angeles Healthcare SystemLos Angeles, CA, USA; ^2^PortlandOR, USA; ^3^Department of Psychology, Trinity Christian College, Palos HeightsIL, USA

**Figure 1:**
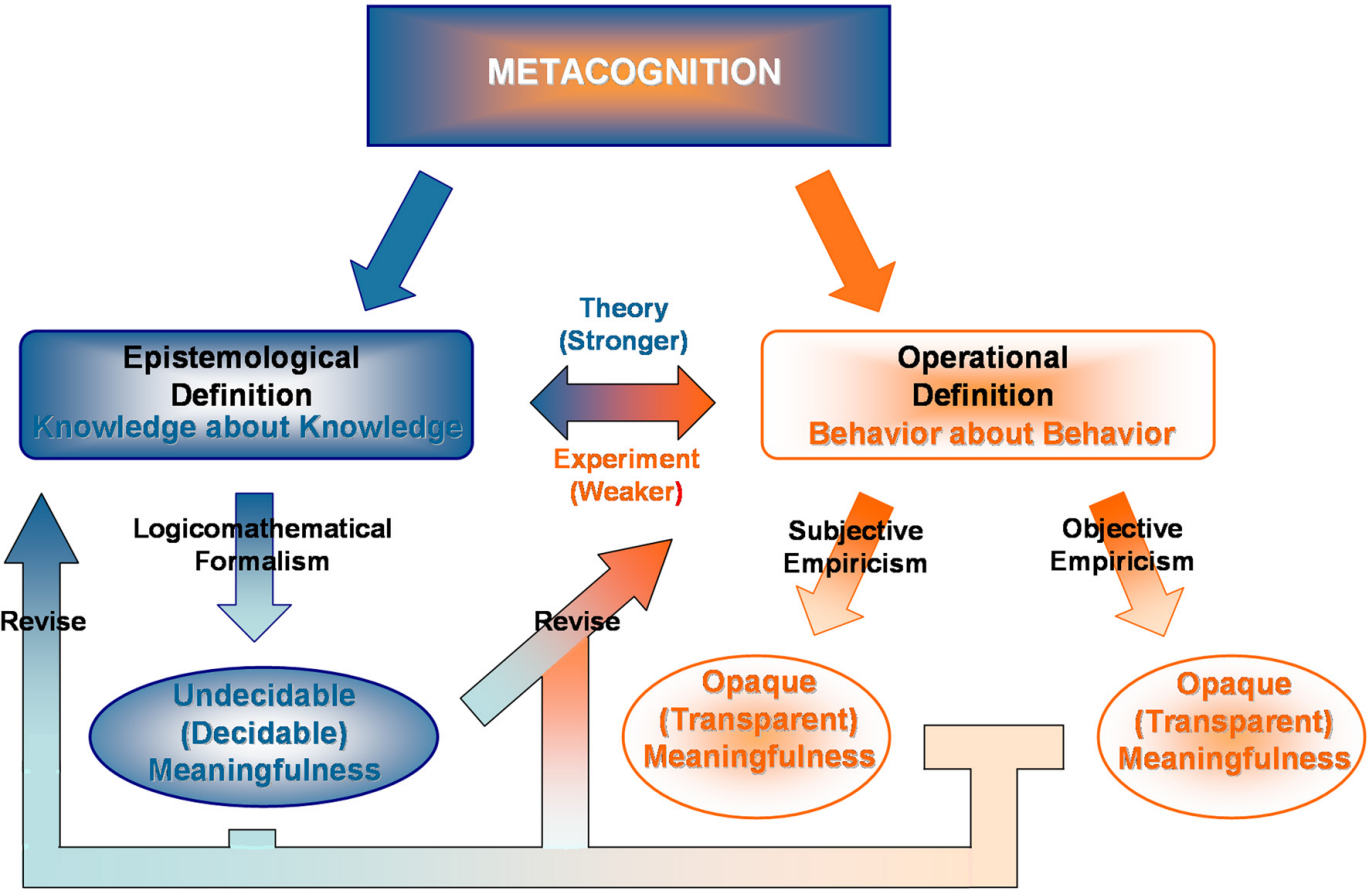
**Diagram outlines relationship of metacognition undecidability and opacity with epistemological and operational definitions, where decidability of logicomathematical formalism is a stronger, more global condition than transparency of empiricism.** Interestingly, the nested referent structure of epistemological and operational definitions may also lead to the paradox that not only is the meaningfulness of metacognition undecidable, but so too is the meaningfulness of (explicit and implicit) reasoning used to reach that conclusion. Anti-mechanistic, -algorithmic, or -logic views of animal and human intelligence find varying degrees of support from noted thinkers in physics (Penrose, 1994), logic (Kreisel, 1967), philosophy, (Lucas, 1962) and psychology (Kahneman et al., 1982). Though a staunch anti-Intuitionist and pro-mathematical Platonist, Gödel (1951/1995) believed the human mind could overcome with empirical certainty such circumstances as “absolutely unsolvable Diophantine problems” and other undecidable problems suggestive of his theorems.

Metacognition, defined either epistemologically as knowledge about knowledge or operationally as behavior about behavior (Koriat, 2007), presumably enables intelligent agents to self-referentially and, in social contexts (Bahrami et al., 2010, 2012), group-referentially monitor and control emotions, moods, perceptions, memories, reasoning, decisions, and actions. At an epistemological level of description, the nested referent nature of metacognition succumbs to problems originating from recursively enumerable propositional logic; that, as Kurt Gödel (1931) first proved for Bertrand Russell and Alfred North Whitehead's axiomatic *Principia Mathematica*, the meaning of statements created about conditions of a system or set of systems by the respective same system or set of systems can be formally undecidable. Momentarily ignoring peripheral confounds introduced by stochastic and imperfect biological systems, animal and human metacognitive operations and all their possibilities must thus exist in a universe of graded logicomathematical consistency (i.e., All theorems are true syntax-correct propositions of the system.) and completeness (i.e., All true syntax-correct propositions of the system are theorems.) (Kreisel, 1967). Because strong consistency excludes strong completeness, the knotted statements of metacognition may show themselves to be falsehoods, truths, or truths essentially unverifiable in theory as well as in subjective and objective practice (Figure 1) (Raattkainen, 2005).

Psychologists well understand the fallibility of formal logic systems and of axiomatic animal and human psychological processes, including, among other phenomena, feature detection, inferential judgments, error diagnosis and correction, concept formation, memory storage and retrieval, and introspection (cf. Nisbett and Ross, 1980; Kahneman et al., 1982; Watanabe and Huber, 2006). We often stipulate—with qualifications—the flawed definition(s) and agent execution of cognition and metacognition (e.g., Shimamura and Metcalfe, 1994; Koriat and Goldsmith, 1996, 1998; Smith, 2009; Terrace and Son, 2009; Frith, 2012; Fleming et al., 2012; Yeung and Summerfield, 2012). For pragmatic reasons, many of us enthusiastic about studying metacognition also avoid the strange, looping causality of self-reference exposed with Gödelian numbering to concentrate on solving basic and/or clinical empirical difficulties that arise when trying to identify this stubbornly opaque hypothetical construct of healthy and pathological minds (e.g., Bach and David, 2006; Koren et al., 2006; Vance, 2006; Carruthers, 2009; Gumley, 2011; Brevers et al., 2013).

The operational opacity of metacognition becomes arguably most apparent when considering: (1) poor subjective accessibility to the cognition and metacognition of animals and humans of limited or no language proficiency (cf. Hampton, 2009; Fleming and Dolan, 2012; Kepecs and Mainen, 2012; Smith et al., 2012), (2) the synergism and antagonism of unreliable explicit and implicit psychological components that mask real metacognitive abilities and capacities from agent and external observer (cf. Hampton, 2009; Fleming et al., 2012), and (3) the occasional independence between metacognition and cognitive skills which may exacerbate the preceding two problems (cf. Koriat and Goldsmith, 1998; Schneider, 1999). A case illustrating these three classes of problems is found for Paulus et al. (2013), who report evidence of implicit metacognition in normal preschool children performing a paired-associates learning and memory task. The authors confront the dilemma of metacognition opacity with a paradigm common to belief, memory, Theory of Mind, and now metacognition research (cf. Nisbett and Ross, 1980; Penn and Povinelli, 2007; Carruthers, 2009; Hampton, 2009; Izard, 2009; Frith, 2012; Skarratt et al., 2012); they prescribe objectively observable, variable primary and secondary behaviors that can be scored for accuracy and/or efficiency and for cross-correlations involving use of secondary behaviors to monitor and control primary behaviors. A typical test scenario has subjects follow associative learning with forced-choice declarative recognition of perceptual/conceptual pairings between two separate images. Subjects subsequently give confidence judgments of memory accuracy through explicit self-reports, such as scalar feelings, and implicit reactions, such as changes in traceable voluntary gaze or involuntary saccades, pupil dilation, pressor effects, and electrical properties of skin. Ratings of accuracy confidence reflect an agent's more-or-less accessible knowledge representation and decisional or post-decisional monitoring and control.

By combining experimental protocols that test for self-reports and behavioral reactions, scientists, including Paulus et al. (2013), hope to objectify a subject's mental states irrespective of his/her language skills as well as dissociate and double dissociate confounding explicit and implicit information processing. For example, Paulus et al. (2013) demonstrate implicit confidence judgments (i.e., gaze direction and duration and pupil area) can be superior in memory accuracy to prompted explicit judgments (i.e., self-report). Increased eye responses coincide with increased (pre)attention demand or load, suggesting humans might be more capable of successfully monitoring judgments at a preattentive or non-conscious level during earlier stages of cognitive development. This sort of approach toward investigating metacognition now seems routine though its widespread appeal dates back roughly 35 years (cf. Flavell, 1979). It builds upon the efforts of Thorndike (1911), Köhler (1927), and additional pioneering psychologists, who inferred mentality in laboratory and wild animals incapable of symbolic communication. Middle to late twentieth century primatologists especially began to embrace behavioral methodology as a tool to compare and contrast the cognitive development and capacity of non-human primates with humans, particularly periverbal neonates, toddlers, and preschoolers (Parker and McKinney, 1999). The value of cross-taxonomic analyses and concomitant realization that oral and written verbal self-reports can be inaccurate, due to lack of awareness and incidental mental events, such as confabulation, illusory perception, cued or irretrievable memory, affect bias, and knowledge lean (e.g., Nisbett and Wilson, 1977; Berry and Broadbent, 1984), triggered this paradigm shift away from once favored subjective human introspection techniques. Some authorities even believe advances in metacognition research are only achievable with improved behavioral models (e.g., Terrace and Son, 2009; Kepecs and Mainen, 2012).

Besides phenomenological and mechanistic insights, studies such as Paulus et al. (2013) on developmental complexity and stages of metacognition across animal and human lifespans figure to clarify the ecological relevance of metacognition, with numerous ramifications for parenting, education, crime, and public health. The weighty private and public consequences of metacognition then necessitate that metacognition be researched and interpreted with exceeding care in regard to cognitive transparency of test paradigms. Debates concerning the effectiveness of standard behavioral tests to reduce metacognition opacity remain spirited at best. Akin to faults of introspection and self-reports (Clark, 2012), behaviors are susceptible to incidental and (pre)attentive disturbances which influence perception, memory, and performance and which may be improperly controlled with experiment conditions (cf. Penn and Povinelli, 2007; Hampton, 2009; Smith, 2009; Smith et al., 2012). Perhaps most alarming, however, are assertions condemning instances of behaviorally measured metacognition in animals and language-challenged humans as nothing more than automatic (i.e., non-reflective), low-level associative response contingencies (Penn and Povinelli, 2007; Hampton, 2009). A conclusion Paulus et al. (2013) also admit could not be negated for some of their observations on the relationship of pupil size, memory retrieval, and metacognition.

This kind of harsh indictment, if correct, despite correlative neurometric findings from non-invasive EEG, PET, fMRI, and SQUID brain recordings (e.g., Fleming and Dolan, 2012), sadly almost reaffirms outcomes of Gödel's Incompleteness Theorems—the nested referential structure of metacognition may prevent metacognition from ever being fully transparent to empiricism and, therefore, makes its emergent meaningfulness perhaps hopelessly undecidable for epistemological and operational definitions (Figure 1). However, empiricists tend to believe, as we and Paulus and colleagues do, perfecting the state-of-art for metacognition paradigms gives fair cause for optimism. Gödel (1951/1995) himself considered logicomathematical formalism to exist independently above animal and human mentality. He also denied that his theorems substantiated, with logicomathematical certainty, the concept of humanly unsolvable objective or subjective problems. With irony, he felt this could be known for external or internal (self) observer alone through rational empiricism.
